# Congenital Hyperinsulinism and Evolution to Sulfonylurearesponsive Diabetes Later in Life due to a Novel Homozygous p.L171F *ABCC8* Mutation

**DOI:** 10.4274/jcrpe.galenos.2018.2018.0077

**Published:** 2019-02-20

**Authors:** Emregül Işık, Hüseyin Demirbilek, Jayne A. Houghton, Sian Ellard, Sarah E. Flanagan, Khalid Hussain

**Affiliations:** 1 Gaziantep Children’s Hospital, Clinics of Paediatric Endocrinology, Gaziantep, Turkey; 2 Hacettepe University Faculty of Medicine, Department of Paediatric Endocrinology, Ankara, Turkey; 3 University of Exeter Medical School, Institute of Biomedical and Clinical Science, Exeter, United Kingdom; 4 Sidra Medical and Research Center, Clinic of Paediatric Medicine, Doha, Qatar

**Keywords:** Congenital hyperinsulinism, MODY, ABCC8 mutation, children

## Abstract

Congenital hyperinsulinism (CHI) is the most common cause of persistent hypoglycemia in infants and children. Recessive inactivating mutations in the *ABCC8* and *KCNJ11* genes account for approximately 50% of all CHI cases. Hyperinsulinaemic hypoglycaemia in infancy and diabetes in later life have been reported in patients with *HNF1A, HNF4A* and *ABCC8* mutations. Herein, we present a child who was diagnosed with CHI at birth, then developed diabetes mellitus at the age of nine years due to a novel homozygous missense, p.L171F (c.511C>T) mutation in exon 4 of *ABCC8*. The parents and one sibling were heterozygous carriers, whilst a younger sibling who had transient neonatal hypoglycemia was homozygous for the mutation. The mother and (maternal) uncle, who was also heterozygous for the mutation, developed diabetes within their third decade of life. The preliminary results of sulphonylurea (SU) treatment was suggestive of SU responsiveness. Patients with homozygous *ABCC8* mutations can present with CHI in the newborn period, the hyperinsulinism can show variability in terms of clinical severity and age at presentation and can cause diabetes later in life. Patients with homozygous *ABCC8* mutations who are managed medically should be followed long-term as they may be at increased risk of developing diabetes after many years.

**What is already known on this topic?**

Homozygous *ABCC8* mutations cause severe, persistent, diffuse hyperinsulinemic hypoglycaemia (HH) which is usually diazoxide unresponsive and requires surgical therapy. In medically managed patients with congenital hyperinsulinism, disease symptoms become milder over time. HH in the neonatal period, and subsequent diabetes, have been reported in heterozygous mutations of *HNF4A* and *HNF1A* as well as heterozygous *ABCC8* mutations.

**What this study adds?**

We describe the first homozygous *ABCC8* mutation with hyperinsulinemic hypoglycaemia (HH) in the neonatal period and its evolution to complete insulin deficient, sulphonylurea responsive diabetes mellitus. Findings from this present work, which show a broad clinical spectrum from asymptomatic to mild symptomatic hypoglycemia and severe hypoglycemia as well as insulin deficient diabetes mellitus in family members with identical mutation confirm the phenotypical variations in *ABCC8* mutations. This present case report emphasizes the need for long-term follow up of patients with HH in the neonatal period due to *ABCC8* mutations, particularly in those who have received medical therapy for risk of developing diabetes in later life.

## Introduction

Adenosine triphosphate (ATP)-sensitive potassium (K_ATP_) channels play an essential role in the regulation of insulin secretion from the pancreatic beta-cell; the key mechanism maintaining the blood glucose level in a narrow range of 3.5-5.5 mmol/L (1,2,3). K_ATP_ channels are open at low glucose levels (1). Increased metabolism, resulting in an increased ATP/adenosine diphosphate ratio, leads to closure of the K_ATP_ channel, depolarisation of the beta cell membrane and subsequent calcium influx through voltage-gated calcium channels. This in turn leads to insulin secretion via the exocytosis of secretory granules (2,3). Dysfunction of the K_ATP_ channel can cause either congenital hyperinsulinism (CHI) or diabetes (neonatal or adult onset) (1,4,5,6,7,8,9). CHI occurs when K_ATP_ channels are absent on the cell membrane or when they remain closed despite low glucose levels. In contrast, diabetes occurs if K_ATP_ channels remain open despite high blood glucose concentrations and increased metabolism in the beta cell (1,4). Recessive inactivating mutations of the K_ATP_ channel genes (*ABCC8* and *KCNJ11*) are the most common cause of severe, diazoxide unresponsive, diffuse CHI which usually requires pancreatectomy (1,10). Patients with dominant mutations of K_ATP_ channel genes, *ABCC8* and *KCNJ11*, may cause variable phenotype ranging from asymptomatic macrosomia, mild diazoxide responsive CHI to severe persistent hyperinsulinaemic hypoglycaemia (HH) as well as diabetes mellitus in later life (7,8,9,11).

CHI within the neonatal period and evolution to diabetes later in life has been reported in individuals with heterozygous inactivating mutations in the hepatocyte nuclear factor 4A and 1A genes (*HNF4A* and *HNF1A*) (12,13,14) and dominant mutations in *ABCC8* genes in a very limited number of cases (1,7,11,13,15,16,17,18,19,20,21). 

To the best of our knowledge, CHI due to homozygous *ABCC8* mutations and evolution to complete insulin deficient-diabetes later in life has not been reported. Herein, we present a patient with a novel, homozygous *ABCC8* mutation who was diagnosed with CHI in the neonatal period and developed diabetes at the age of nine years.

## Case Report

**Figure 1:**
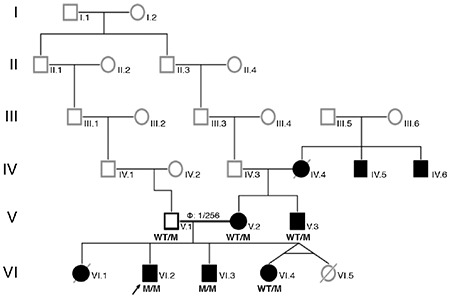
Pedigree of the family. The members developed either hypoglycaemia, diabetes or both are indicated as affected and shown with black-filled boxes. IV.4: Insulin dependent diabetes since 35 years-old, developed diabetic nephropathy (chronic renal failure) (reportedly), IV.5: Had insulin dependent diabetes and diabetic nephropathy (reportedly), IV.6: Diabetes and bilateral visual loss was reported, V.1: Father, 41 years old, apparently healthy with normal glucose and HbA1c (5.6%) levels, V.2: Mother 37 years old, developed insulin dependent diabetes during pregnancy and has been on insulin treatment since 24 years old, changing the treatment to SU therapy is in progress (see the section of the case report concerning sulphonylurea treatment), V.3: 40 years old, had insulin dependent diabetes mellitus since 32 years-old, VI.1: Born at term, macrosomic birth weight (4750 gram; 2.8 SDS), hypoglycaemia was detected during the neonatal period, died at 1-month during hospitalization with unknown etiology, VI.2: Index patient, VI.3: 9.5 years-old, born at term, birth weight was 3750 gram (0.4 SDS), had transient hypoglycemia during the neonatal period, latest blood glucose and HbA1c levels were normal, VI.4: 6.5 years old, born at seven months of gestation from a twin pregnancy, birth weight was 1250 gram (1.05 SD), HbA1c is normal M: mutated, WT: wild type, SDS: standard deviation score, SU: sulphonylurea, HbA1c: haemoglobin A1c

A nine year-old Turkish boy (VI.2 in Figure 1) presented with abdominal pain and fever. He was diagnosed with perforated appendicitis and was referred to the endocrine clinic for coexisting hyperglycaemia (blood glucose level was 27.75 mmol/L). A detailed family history revealed the presence of diabetes in multiple members of the maternal family (see details on the pedigree and footnotes). Specifically, the patient’s mother was on insulin therapy for diabetes mellitus which had been diagnosed during the first trimester of pregnancy, when she was 24 years of age. A maternal uncle was also affected. There was also a history of neonatal hypoglycaemia of varying duration and severity affecting two of the patient’s siblings.

The patient was the first living child of the family and was born with a birth weight of 3750 grams (+6.6 SD) via caesarian section at a gestation age of 29 weeks. Parents were distantly related. He presented with a hypoglycaemic episode at postnatal day one (blood glucose was 1.33 mmol/L and simultaneous insulin level was 22.7 µIU/mL, C-peptide 5.42 ng/mL (0.9-7.1). A diagnosis of HH was considered and diazoxide was commenced. The patient developed pulmonary edema, which was considered likely to be a complication of treatment with diazoxide. Diazoxide was subsequently stopped and octreotide therapy was introduced. Hypoglycaemia remitted at the age of three months and the child remained free of hypoglycaemic episodes until nine years of age, when he was admitted to our hospital.

On admisson, the child was lethargic and had pale and grayish colour skin. His height was 140 cm [0.7 standard deviation score (SDS)], weight was 35 kg (0.8 SDS), and body mass index (BMI) 17.8 (0.7 SDS). Respiratory rate was 20 breaths/minute, heart rate was 72 beats/minute and blood pressure was 90/60 mmHg. There was abdominal distention, rigidity and rebound tenderness on physical examination. The patient underwent emergency appendectomy. During the post-operative period hyperglycaemia persisted and subcutaneous insulin therapy was introduced. Laboratory investigations revealed a blood glucose of 13.2 mmol/L with a simultaneous insulin of 8.82 µIU/mL (2.6-25), C-peptide: 1.28 ng/mL (0.9-7.1). Glycosylated haemoglobin A1c (HbA1c) was 9.1% (76 mmol/L). Islet cell, anti-insulin and anti-glutamic acid decarboxylase antibodies were negative. Over the following two months the insulin requirement gradually decreased until insulin treatment could be completely withdrawn. During the follow-up, HbA1c remained within the range of the high normal limits (6.2% to 6.4%) with dietary intervention and lifestyle changes. At the age of 11.5 years HbA1c was shown to be again significantly raised at 9.6% (81 mmol/L). At this time his weight was 46 kg (0.9 SDS), height was 152 cm (0.8 SDS) and BMI was 19.9 (0.9 SDS). The patient and family refused recommencement of insulin therapy. Subsequently, HbA1c increased to 11.4% (101 mmol/l) at the age of 12 years when an oral glucose tolerance test suggested insulin deficient-diabetes mellitus (Table 1).

### Genetic Testing

Genomic DNA was extracted from peripheral leukocytes using standard procedures and the coding regions and intron/exon boundaries of the *ABCC8*, *KCNJ11*, *HNF4A* and *HADH* genes were amplified by polymerase chain reaction (primers available on request). Amplicons were sequenced using the BigDye Terminator Cycle Sequencing Kit V.3.1 (Applied Biosystems, Warrington, UK) according to manufacturer’s instruction and reactions were analysed on an ABI 3730 Capillary sequencer (Applied Biosystems, Warrington, UK). Sequences were compared with the reference sequences (NM_001287174.1, NM_000525.3, NM_175914.4 and NM_005327.4) using Mutation Surveyor v5.0.1 software (SoftGenetics, State College, Pennsylvania, USA). The variant was classified using the American College of Medical Genetic and Genomics/Association for Molecular Pathology guidelines (22).

A written informed consent was obtained from the patients and/or their legal guardians.

## Results

**Figure 2:**
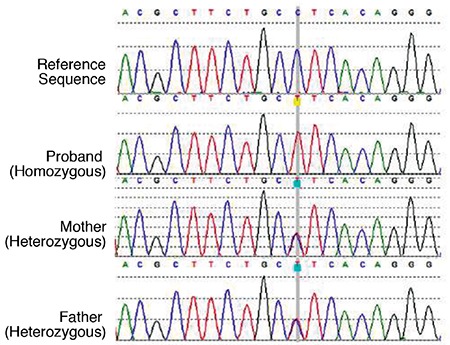
Electropherograms of the reference, index case and parents for c.511C>T (p.L171F) mutation

The index patient (VI.2, see Figure 1) was found to be homozygous for a novel missense c.511C>T (p.L171F) variant in exon 4 of *ABCC8* (Figure 2). The p.L171F variant affects a highly conserved amino acid and *in silico* analysis predicted the variant to be disease-causing (Alamut Visual V2.10 Software, Rouen, France). Mutation testing showed that the variant co-segregated with diabetes and hypoglycemia within the family, with an incomplete penetrance of heterozygous carriers (Figure 1).

### Treatment and Follow-up

Following detection of the *ABCC8* mutation, a trial of sulphonylurea (SU) treatment was commenced in the index case and his mother, who had been on insulin therapy for 13 years in an outpatient setting (Table 2). The mother’s daily insulin dose requirement was reduced by approximately 50% from the baseline at the first week of the SU therapy with improved blood glucose measurements. The index case also responded to SU therapy and even developed one hypoglycaemic episode following SU therapy. Although the SU doses were adjusted accordingly, the family avoided giving the glibenclamide regularly due to the severe hypoglycaemic episodes which had not been observed while he was on insulin therapy or during fasting.

## Discussion

Herein, we present a patient with a novel homozygous *ABCC8* mutation who was diagnosed with HH in the neonatal period and diabetes at the age of nine years. Hyperglycemia was first recognized during acute appendicitis which suggested stress-induced hyperglycemia. However, the patient had persistent hyperglycemia which required insulin therapy, a history of HH and relatives with autoantibody-negative diabetes. These findings were suggestive of monogenic diabetes, which was confirmed by molecular genetics analysis.

Homozygous K_ATP_ channel gene mutations are the most common cause of severe, diazoxide unresponsive HH which often requires pancreatectomy (3,13). However, clinical heterogeneity is observed in patients with dominant *ABCC8* mutations (9). Kapoor et al (23) reported a marked clinical heterogenity in siblings with identical mutations in *ABCC8*, ranging from asymptomatic hypoglycaemia to macrosomia, transient HH or severe HH and development of diabetes mellitus later in life. Besides, a heterogeneous nature is observed regarding severity and response to medical treatment and age of onset of symptoms (23).

Variations in the severity of HH and clinical course have also been reported in a mother and her daughter with a heterozygous *E1506K* mutation in *ABCC8* (20). In this report the child had severe symptoms and hypoglycaemic convulsions at age three months while the mother had subtle symptoms of hypoglycaemia followed by gestational diabetes which persisted after delivery. 

Similarly, a marked clinical heterogenity was also observed in this present family. While one of the homozygous siblings (VI.2, index case) had prolonged HH and required medical therapy, the other sibling with an identical homozygous mutation (VI.3) suffered from transient hypoglycemia in the first week of life which remitted within three months. Diabetes was observed in the heterozygous mother and a maternal uncle, but the father (V.1) who was also heterozygous for the *ABCC8* mutation, had normal fasting plasma glucose and HbA1c levels. Unfortunately, the family refused performance of oral glucose tolerance test in the individuals who carried the mutation, but had not yet developed fasting hyperglycaemia or elevated HbA1c. We could also not perform genetic analysis in other relatives who also had diabetes with renal and ocular complications. We, therefore, were not able to confirm whether the diabetes in these additional family members was due to the *ABCC8* mutation.

Indeed, the clinical course for patients with *ABCC8* mutations is also substantially variable (1,7,13,15,16,17,18,19,20). Clinical features include hypoglycaemia within the neonatal period which remits over time, coexistence of hypoglycaemia and post-fed hyperglycaemic episodes, impaired fasting glucose or impaired glucose tolerance in response to a glucose load and in a few cases the development of diabetes (1,7,11,13,15,16,17,18,19,20). Regarding the type of mutation reported in *ABCC8* which caused neonatal HH and diabetes later in life, only a few cases were reported to have a homozygous mutation (18), whilst the majority had heterozygous or compound heterozygous mutations (7,11,15,17,19,20). 

Biallelic (either homozygous or compound heterozygous) *ABCC8* mutations usually cause severe, diazoxide unresponsive HH which often requires surgical management (3). Therefore, the number of medically managed cases, particularly those with long-term follow-up, is very small. This limits our understanding of the underlying mechanisms and experience in the management of cases who develop hyperglycemia later in life. The data from the reported cases and experimental studies suggest that the key mechanisms are dysregulated insulin secretion, impaired first phase insulin secretion, delayed insulin response and b-cell apoptosis mediated via enhanced b-cell depolarisation, resulting in increased calcium ion entry into the cell (7,8,9,13,15,16,17,18,19,20,24,25,26). 

Taking into account the previously reported cases, our patient is the only case with a homozygous *ABCC8* mutation who presented with CHI (confirmed by clinical and biochemical evidence and mutation analysis) within the neonatal period which evolved into complete insulin deficient diabetes later in life. Therefore, this family provides novel insights into the clinical heterogeneity of CHI and later onset diabetes in patients with homozygous *ABCC8* mutations.

Neonatal diabetes due to a dominant activating mutation of a K_ATP_ channel gene (*KCNJ11* or *ABCC8*) is usually sulphonylurea responsive (27,28). We also performed a trial of SU therapy in the index case and his heterozygous mother who had insulin dependent diabetes. Preliminary results suggested a favorable SU responsiveness. Since SU drugs work by binding to the SUR1 subunit of the K_ATP_ channel, a positive response to SU therapy suggested that the presence of a homozygous mutation may not completely abolish the channel function.

In conclusion, we present the novel missense c.511C>T (p.L171F) *ABCC8* mutation causing neonatal HH and SU-responsive diabetes mellitus later in life. There are, however, some limitations in interpreting the phenotype-genotype relationships observed in this family. Firstly, we were not able to analyse the mutation status of other family members with diabetes mellitus. Secondly, although clinical evidences and bioinformatic tools confirmed the pathogenicity of the novel mutation, functional analyses have not been undertaken to assess the role of the variant *in vitro*. These results highlight the need for the long-term follow up of a larger series of CHI patients with homozygous *ABCC8* mutations who have been managed medically. In addition further evaluation of these variants, including functional analysis, to better understand the underlying molecular mechanism and phenotype-genotype relationships should be performed.

## Figures and Tables

**Table 1:**
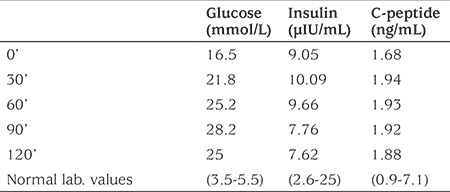
Oral glucose tolerance test results of index case at age 12 years

**Table 2:**
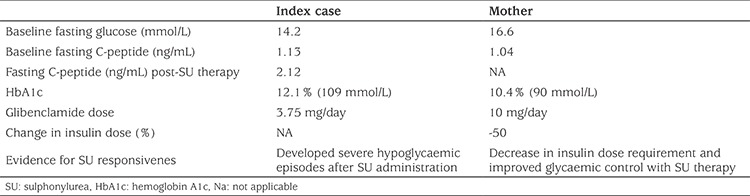
Sulphonlyurea treatment trial results in the index case and his heterozygous carrier mother with diabetes mellitus
